# Multilayer 3D bioprinting and complex mechanical properties of alginate-gelatin mesostructures

**DOI:** 10.1038/s41598-023-38323-2

**Published:** 2023-07-12

**Authors:** Anahita Ahmadi Soufivand, Jessica Faber, Jan Hinrichsen, Silvia Budday

**Affiliations:** Institute of Continuum Mechanics and Biomechanics, Department of Mechanical Engineering, Friedrich-Alexander-Universität Erlangen-Nürnberg, 91058 Erlangen, Germany

**Keywords:** Biomedical engineering, Implants, Tissue engineering, Gels and hydrogels, Rheology

## Abstract

In the biomedical field, extrusion-based 3D bioprinting has emerged as a promising technique to fabricate tissue replacements. However, a main challenge is to find suitable bioinks and reproducible procedures that ensure good printability and generate final printed constructs with high shape fidelity, similarity to the designed model, and controllable mechanical properties. In this study, our main goal is to 3D print multilayered structures from alginate-gelatin (AG) hydrogels and to quantify their complex mechanical properties with particular focus on the effects of the extrusion process and geometrical parameters, i.e. different mesostructures and macroporosities. We first introduce a procedure including a pre-cooling step and optimized printing parameters to control and improve the printability of AG hydrogels based on rheological tests and printability studies. Through this procedure, we significantly improve the printability and flow stability of AG hydrogels and successfully fabricate well-defined constructs similar to our design models. Our subsequent complex mechanical analyses highlight that the extrusion process and the mesostructure, characterized by pore size, layer height and filament diameter, significantly change the complex mechanical response of printed constructs. The presented approach and the corresponding results have important implications for future 3D bioprinting applications when aiming to produce replacements with good structural integrity and defined mechanical properties similar to the native tissue, especially in soft tissue engineering. The approach is also applicable to the printing of gelatin-based hydrogels with different accompanying materials, concentrations, or cells.

## Introduction

3D printing is a promising technology to produce complex structures layer by layer^1–4^. It becomes feasible to fabricate complicated geometries that are impossible to obtain through conventional manufacturing methods^5–8^. 3D bioprinting applies this technique in the biomedical field by deposition of cells and biomaterials in a predefined path to fabricate tissue replacements^9–12^. These replacements should have similar mechanical properties to the native tissue to achieve the appropriate function in realistic physiological loading conditions in the body^13–15^. In addition, the bioprinted structure should have a specific mesostructure, e.g. with a certain pore size, to facilitate nutrition delivery and cellular activities^6,10^. The mesostructure in turn can significantly affect the mechanical properties of printed constructs due to different load-bearing patterns^8^. Consequently, it can also be used to tune the mechanical properties towards a targeted tissue replacement. The effect of the mesostructure generated through certain print patterns has hardly been studied due to difficulties associated with bioprinting constructs with enough structural integrity^16,17^. Consequently, it is essential to ensure high printability and shape fidelity of the hydrogel to tackle these issues.

Among different bioprinting techniques, microextrusion bioprinting was used widely in the biofabrication field using a broad range of biomaterials and bioinks^18–20^. In microextrusion bioprinting, the bioink is extruded from the nozzle through the applied pressure in the syringe^6,10^. During the extrusion process, the viscous bioink is exposed to shear stresses that may affect the material properties of the printed construct^21^. Therefore, it is important to investigate how the extrusion affects the mechanical properties of the printed construct, especially with regard to viscoelastic material properties that critically affect the cellular activity.

A first key requirement to bioprint a tissue construct is a biocompatible and printable hydrogel as bioink^22–25^. In the literature, several bioinks, such as gelatin, alginate, hyaluronic acid, gelatin methacrylate, or extracellular matrix (ECM)-based, have been used, both individually and in composite forms^25–28^. Among them, we will here focus on a composite of gelatin and alginate. Gelatin inhibits thermo-gelling properties, which allows us to print structures with good shape fidelity^29,30^. Alginate, on the other hand, introduces crosslinking properties in our bioink and also enhances the printability of the hydrogel^31^. Notably, alginate-gelatin (AG) hydrogels have been widely used as they provide a cell-friendly environment and are easy to prepare and use^31–33^. However, their flow properties during the bioprinting process depend on several parameters, e.g. the individual components’ concentrations, temperature, and time^29,31^. Therefore, maintaining a steady flow for a period of time long enough to fabricate 3D structures is highly challenging. As a result, most previous studies have focused on 2D porous constructs of AG hydrogels^17,29^.

The gelation of AG bioinks and reaching a state of stable rheological properties takes a long time^29^, while the printing process needs to be performed fast^6,10^. In this respect, utilizing a cooling step can be beneficial to decrease the gelation time and fluctuations in the flow rate^34^. Furthermore, as the printability of an AG bioink depends on the nozzle temperature^29^, it is essential to consider this effect when choosing an appropriate temperature for the bioprinting process. Finally, a steady mass extrusion (feed rate) during printing is important to avoid deviation of the printed filament diameter from its design value and to fabricate 3D printed structures geometrically similar to the computer-aided design (CAD) model. Only when carefully considering these aspects, it becomes feasible to print samples with different designs and mesostructures using microextrusion. To the best of our knowledge, there is to date no report on the precise printing of different mesostructures with AG bioinks.

**Figure 1:**
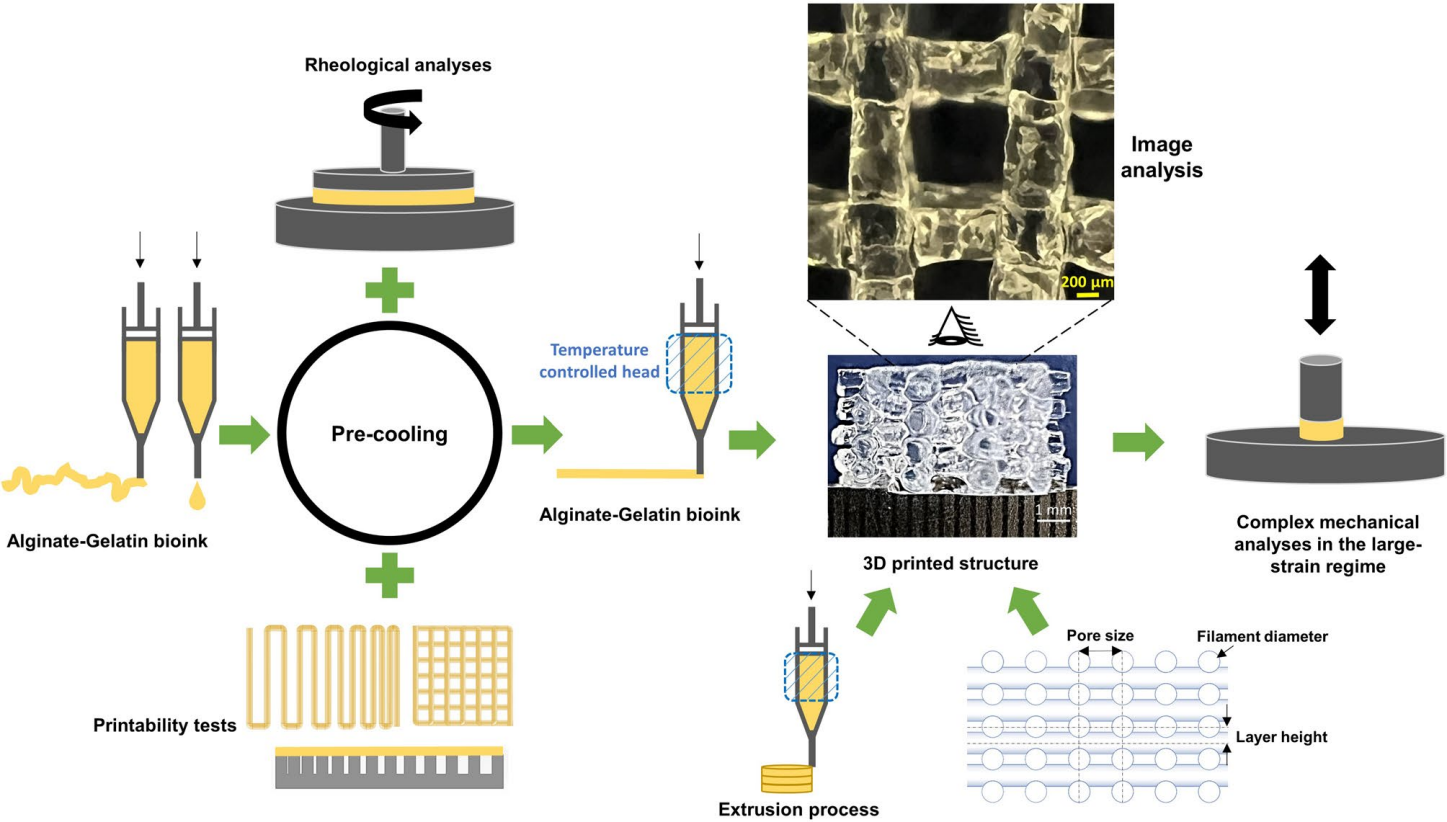
Schematic diagram of the essential steps used to optimize the printing process: Using a pre-cooling step in combination with rheological measurements and printability tests led to highly printable AG bioinks to fabricate different 3D constructs with variable mesostructures and viscoelastic properties.

In this study, we aimed to fabricate well-defined printed structures to investigate the effect of the extrusion process and different mesostructures on the final mechanical properties of the 3D printed constructs. We develop an improved and reproducible procedure for the bioprinting of AG bioinks. Based on rheological measurements and printability studies, we include a cooling step and identify appropriate printing parameters. Like this, we succeed in printing multilayered samples with high control of geometrical parameters. In a next step, we mechanically characterize and compare molded and printed cylindrical samples to investigate the effect of extrusion on the resulting mechanical properties. Finally, we fabricate hydrogel constructs with different filament diameters, pore sizes, and layer heights to evaluate the effect of different mesostructures on the final constructs’ complex response during cyclic compression-tension and stress relaxation loading (Fig. 1).

## Materials and methods

### Bioink preparation

Alginate (type PH163 S2) was purchased from Vivapharm, JRS PHARMA GmbH & Co. KG, and gelatin (type A, 300 bloom derived from porcine skin) was purchased from Sigma Aldrich (Germany). We prepared the AG bioink with 2% (w/v) and 5% (w/v) ratios of alginate and gelatin, respectively, using the method previously described in^31^. Briefly, 600 mg gelatin was first dissolved in 12 ml Dulbecco’s Phosphate Buffered Saline (DPBS, ThermoFisher, Invitrogen, Germany) on a rotational shaker at 37 °C for 1 h. 240 mg Sodium alginate was then added to the gelatin solution, and gelatin–alginate solution was mixed on a rotational shaker at 37°C for an additional 3 hrs. Afterwards, the bioink was kept at 37 °C until further use.

### Rheology

We performed the rheological measurements using the Discovery HR-3 rheometer (TA Instruments, New Castle, USA) with a plate-plate geometry (diameter 40 mm). We placed about 1 ml of the bioink on the bottom plate of the rheometer. By lowering the top plate with a diameter of 40 mm, a thin film of bioink with a thickness of 0.5 mm was generated. We set the frequency to 10 rad/s and strain to 1% to measure in the linear viscoelastic regime. We measured the time-dependent shear storage modulus G’, shear loss modulus G”, complex viscosity ${\eta }^{*}$, and loss tangent tan (δ) by time sweeps (oscillation). All rheological measurements were conducted in triplicate.

### Printability tests

We examined the proper gelation of the bioink initially by extruding it at the required pressure to have a continuous flow. Then, we printed two layers of crossed patterns. For an ideal gelation condition or perfect printability status, the interconnected channels of the constructs would exhibit a square shape. In this condition, the bioink printability (P_r_) value is one^29^, which is defined as:

1 $${P}_{r}=\frac{{L}^{2}}{16A},$$

where L and A denote perimeter and area, respectively. The larger the P_r_ value was, the greater the gelation degree of the bioink was determined to be. The smaller the P_r_ value was, the smaller the gelation degree the bioink was. To determine the P_r_ value, we analyzed the optical images of printed constructs using the ImageJ software to determine the perimeter and area of interconnected channels (n=5).

The minimum required gap between two adjacent filaments was determined by the fusion test^16^. We designed an appropriate pattern with different gaps and analyzed the optical images of the printed bioink to determine the minimum gap to avoid any fusion during printing.

In addition, we measured the resistance of the bioink against the gravity force through collapse tests^30^. We fabricated an artifact to resemble the various possible gaps between two filaments during printing. For each printing condition, we analyzed the optical images of filaments for any collapse occurrence.

### Sample design and fabrication

We designed the samples using SolidWorks 2019 (Dassault Systemes) and then converted the CAD files into printing files using Slic3r, a 3D printing software. On the BioX bioprinter (BICO, Sweden) interface, we set the printing parameters, e.g., nozzle diameter and layer height, according to the design parameters. We prepared five macroporous samples by fabricating larger samples and then extracting cylindrical samples by using a surgical punch with a diameter of 8 mm to eliminate boundary effects during subsequent mechanical testing. In addition, we prepared molded samples using a silicone mold containing holes with a diameter of 8 mm and a height of 4 mm. After fabrication and for crosslinking, we placed the samples in 0.1 M CaCl_2_ solution for about 10 mins. Then, we washed the samples with Hanks’ Balanced Salt Solution (HBSS) purchased from ThermoFisher, Invitrogen, Germany.

### Bioprinting process

We transferred 3 ml of the AG hydrogel to the 3 ml nozzle and placed the nozzle in the centrifuge (Eppendorf 5702, Germany) for 3 mins at 3000 rpm to remove air bubbles. To pre-cool the AG hydrogel and accelerate the gelation process, we then stored the filled nozzle at 4 °C in the refrigerator for 5 mins. Finally, we placed it in the temperature-controlled head of the BioX bioprinter and started to print.

### Characterization of bioprinted constructs

To characterize bioprinted samples, we weighed them to calculate their porosity. To this end, we first determined the density of the bioink as the mass of 3 ml divided by its volume. Then, by knowing the density and sample weight, we obtained the porosity of the sample as:

2 $${P}_{3DP}=\left(1-\frac{M}{\rho {V}_{Bulk}}\right)\times 100\%,$$

where $M$ is sample mass, $\rho$ is the AG bioink density and ${V}_{Bulk}$ is the bulk volume of the sample without porosity^8^. In addition, we calculated CAD and theoretical porosity using:

3 $${P}_{CAD}=\left(1-\frac{{V}_{CAD}}{{V}_{Bulk}}\right)\times 100\%,$$

4 $${P}_{Theory}=\left(1-\frac{{V}_{Theory}}{{V}_{Bulk}}\right)\times 100\%,$$

where ${V}_{CAD}$ and ${V}_{Theory}$ are the occupied volumes in the CAD model with and without layer penetration, respectively. The theoretical porosity was estimated by considering the contact between the filaments of different layers to be only superficial. For more details, we refer to^8^. Afterward, we captured optical images and analyzed them by using the ImageJ software to determine the geometrical parameters (n=5).

Moreover, we calculated the structural integrity of the samples by dividing the printed sample height by the designed height. The designed sample heights ranged from 3.9 to 4.18 mm, depending on their mesostructures. We used the open-source ImageJ software to determine the sample height by measuring the top and bottom layers’ distance from the middle and sides of each sample (n=3).

Finally, we performed cyclic mechanical measurements on our samples to characterize the complex and time-dependent behavior in the large-strain regime^35,36^. A Discovery HR-3 rheometer (TA instruments, New Castle, Delaware, USA) with an 8 mm diameter parallel geometry was used. After calibration of the testing device, we glued an 8 mm circular piece of fine sandpaper to the top geometry and heating plate. Afterwards we carefully placed the sample on a spatula and attached it to the top geometry using an instant adhesive. This procedure significantly improved the adhesion between sample and geometry. Before lowering the top geometry to glue the sample to the heat plate with a preload <0.1 N, we inserted a transparent immersion cup. After a few seconds of waiting time for drying glue, we immersed the sample in a HBSS bath to avoid dehydration during testing. At first, we performed cyclic compression-tension tests with three loading cycles, minimum and maximum stretches of 0.85 and 1.15 and a loading rate of 40 µm/s. Subsequently, stress relaxation tests in compression and tension were conducted at the same minimum and maximum stretches, a loading rate of 100 µm/s and a holding time of 300 seconds. To mimic *in vivo* conditions, all tests were performed at 37°C.

### Statistics

Data were analyzed with a Student’s t-test or one-way ANOVA using the GraphPad Prism software version 8.0 (GraphPad Software, Inc., La Jolla, CA). *p* < 0.05 was considered statistically significant.

## Results

### Effect of the cooling step on the rheological properties of AG bioinks

**Figure 2:**
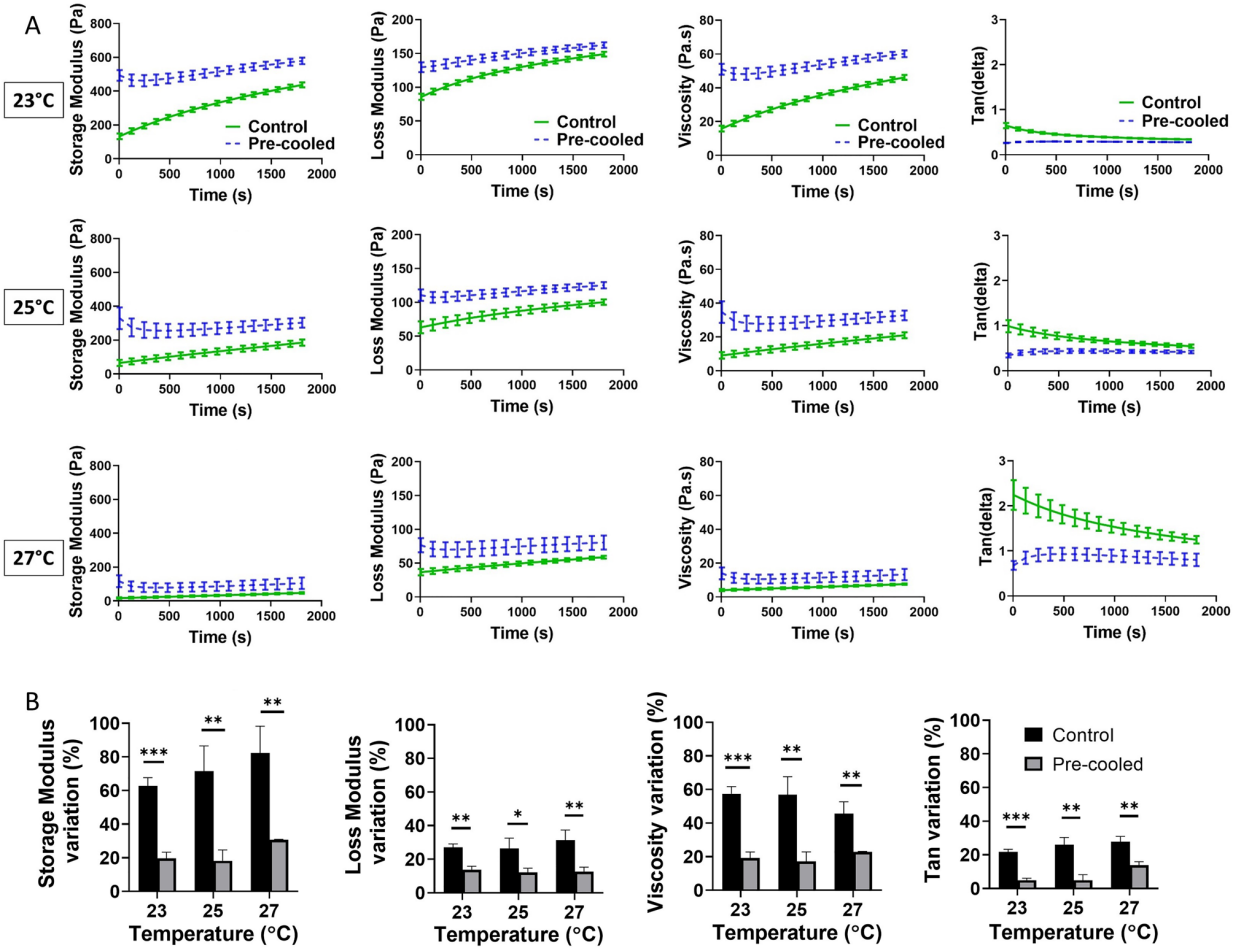
Effect of the cooling step on the rheological properties of AG bioinks: (**A**) Storage and loss moduli, viscosity, and tan (delta) of the 23 °C, 25 °C, and 27 °C; (**B**) Variations of rheological parameters at 20 mins of testing with and without the pre-cooling step at different temperatures. Significance value *p < 0.05, **p < 0.01, ***p < 0.001.

**Figure 3:**
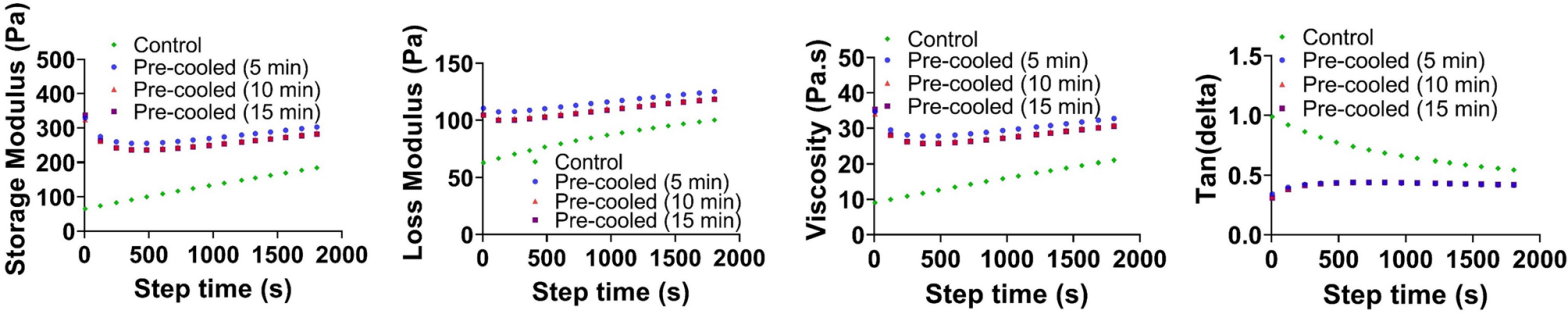
Effect of the cooling time (5, 10 and 15 mins) on the rheological properties of the AG bioink.

Figure 2A demonstrates the effect of the cooling step, in which the bioink was kept at 4 °C for 5 mins, on the rheological properties, i.e. G’, G”, ${\eta }^{*}$, and tan (δ), at three different temperatures (23 °C, 25 °C and 27 °C). Interestingly, the cooling step results in a lower variation in rheological properties during the 30 mins of testing. The effect of pre-cooling becomes even more noticeable for lower temperatures, where the cooling process seems to be more effective. In addition, the cooling step stabilizes the tan (δ), which significantly improves the printability of the AG bioink^31^. To quantify the effect of the cooling step on the rheological properties compared to our control samples, we evaluated the variation of their final value at 20 mins of testing (Fig. 2B). The cooling step significantly stabilizes the rheological properties of the bioink in comparison to no treatment. In other words, through bioink cooling before fabrication, we achieve a more stable printability during the printing process. In a second step, we varied the cooling time to identify the appropriate time for the cooling step resulting in optimal rheological properties (Fig. 3). Our results show that 5 mins of cooling is enough: further increasing the cooling time does not affect the rheological properties.

### Printability and gel stability

**Figure 4:**
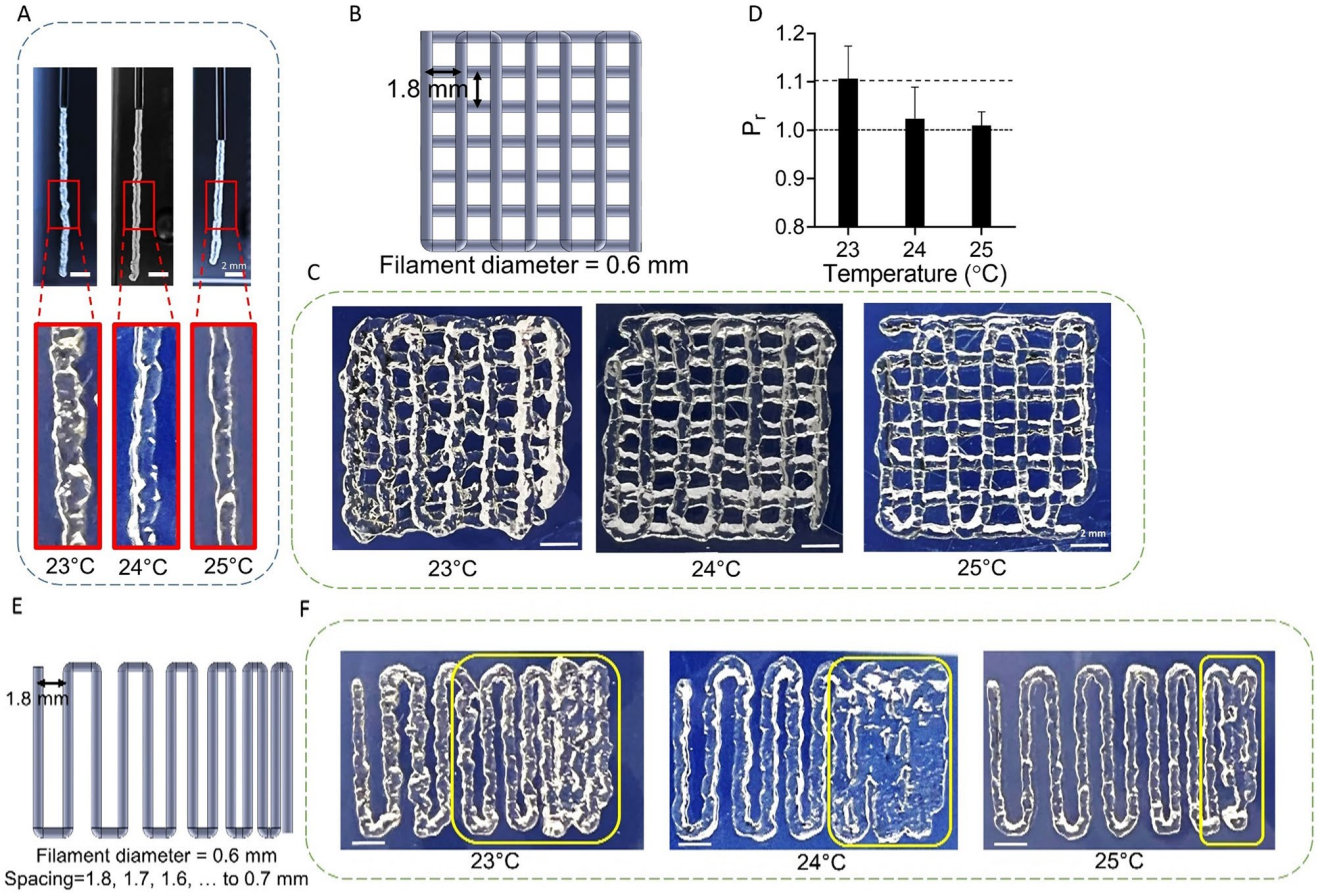
Printability tests of the AG bioink: (**A**) Extruded bioink at 23 °C, 24 °C, and 25 °C; (**B**–**D**) Two layers of bioink for the calculation of the P_r_ value based on the designed pattern; (**E**,**F**) Fusion of filaments during printing at different temperatures.

The variations in rheological properties are lower at 23 °C and 25 °C than at 27 °C, as shown in Fig. 2. To find the suitable temperature for printing, we therefore investigated the printability of the bioink in this range, i.e. at 23 °C, 24 °C, and 25 °C. First, we checked the uniformity of the extruded bioink (Fig. 4A) and we noticed that the uniformity of the filament is higher at 25 °C. Then, we printed two layers of bioink for the calculation of the P_r_ (see Eq. (1)) value based on the designed pattern (Fig. 4B). The corresponding images and P_r_ values are depicted in Fig. 4C andD, respectively. By increasing the temperature, the P_r_ value approaches one so that the printability improves. To additionally study the fusion of filaments during printing for different temperatures, we printed the designed pattern shown in Fig. 4E. We found that the printing resolution increased for higher temperatures. Therefore, based on these printability tests, we chose 25 °C for printing to achieve a better P_r_ value and resolution.

**Figure 5:**
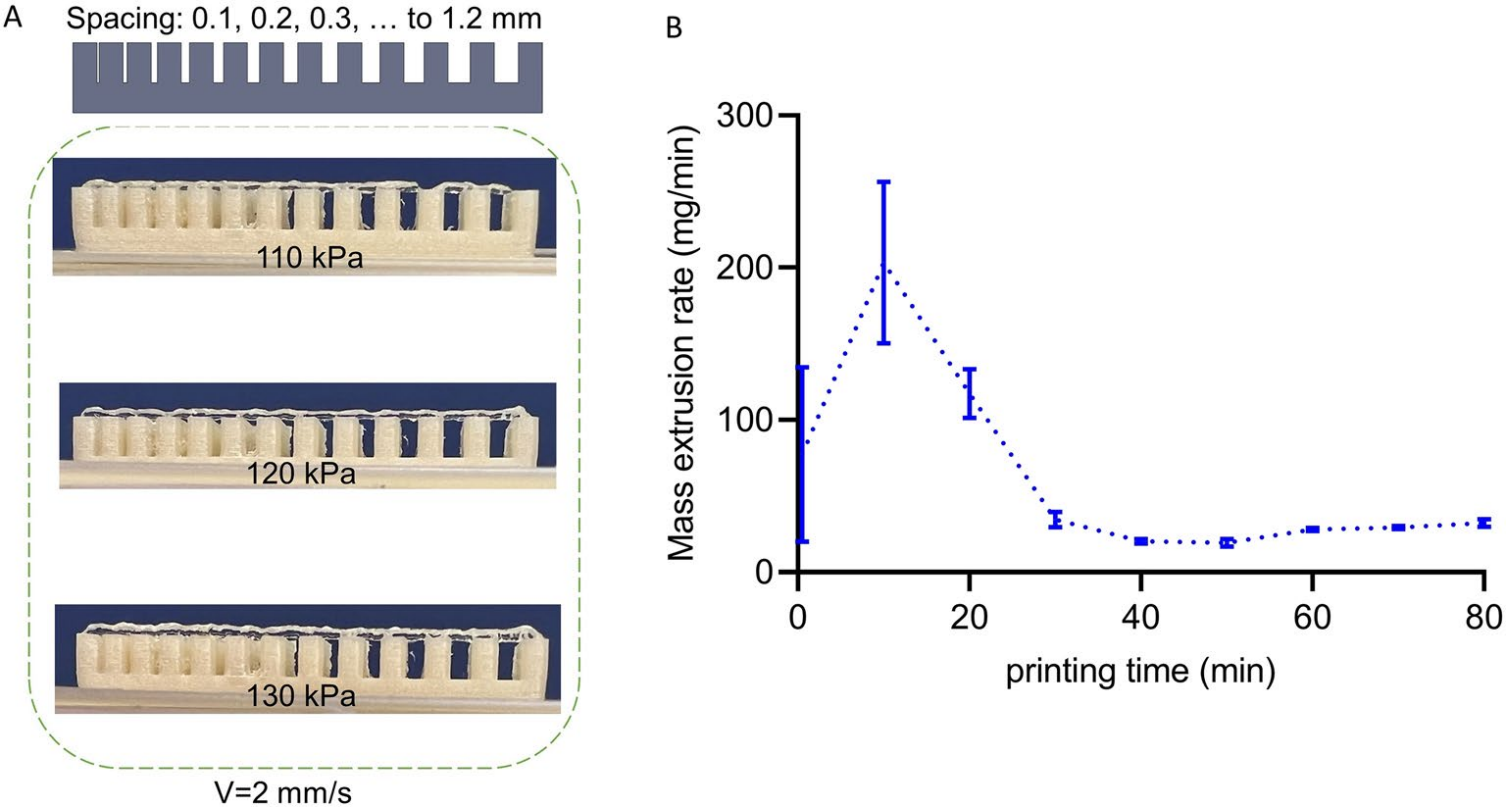
Bioink stability: (**A**) Collapse test to evaluate the resistance against gravity at 25 °C and 110, 120 and 130 kPa pressures; (**B**) Mass extrusion stability of the bioink at 25 °C during the printing time.

Finally, we examined the bioink stability against gravity at 25 °C with the collapse test using the fabricated construct shown in Fig. 5A. We observed that there were no deflections in the filament, even at 1.2 mm spacing between two adjacent underlying filaments at three different printing pressures. Moreover, we measured the mass extrusion rate during the printing time at a constant pressure of 125 kPa (Fig. 5B). We noticed that there were initial fluctuations that vanished after around 40 mins. This initial unstable mass extrusion rate can be associated with the rheological behavior of the material shown in Fig. 2A, where the viscosity initially decreases for the pre-cooled AG bioink at 25 °C. The lower viscosity leads to a higher mass extrusion rate in the nozzle in accordance with the results in Fig. 5B. To avoid such fluctuations for all remaining experiments, we waited 60 mins before starting to print our constructs. This way, we minimized the filament diameter variations throughout the mesostructures while keeping the extrusion pressure constant. Having a constant extrusion flow was essential for our study as we aimed to print structures that resembled the CAD designs as close as possible.

### Effects of printing parameters

**Figure 6:**
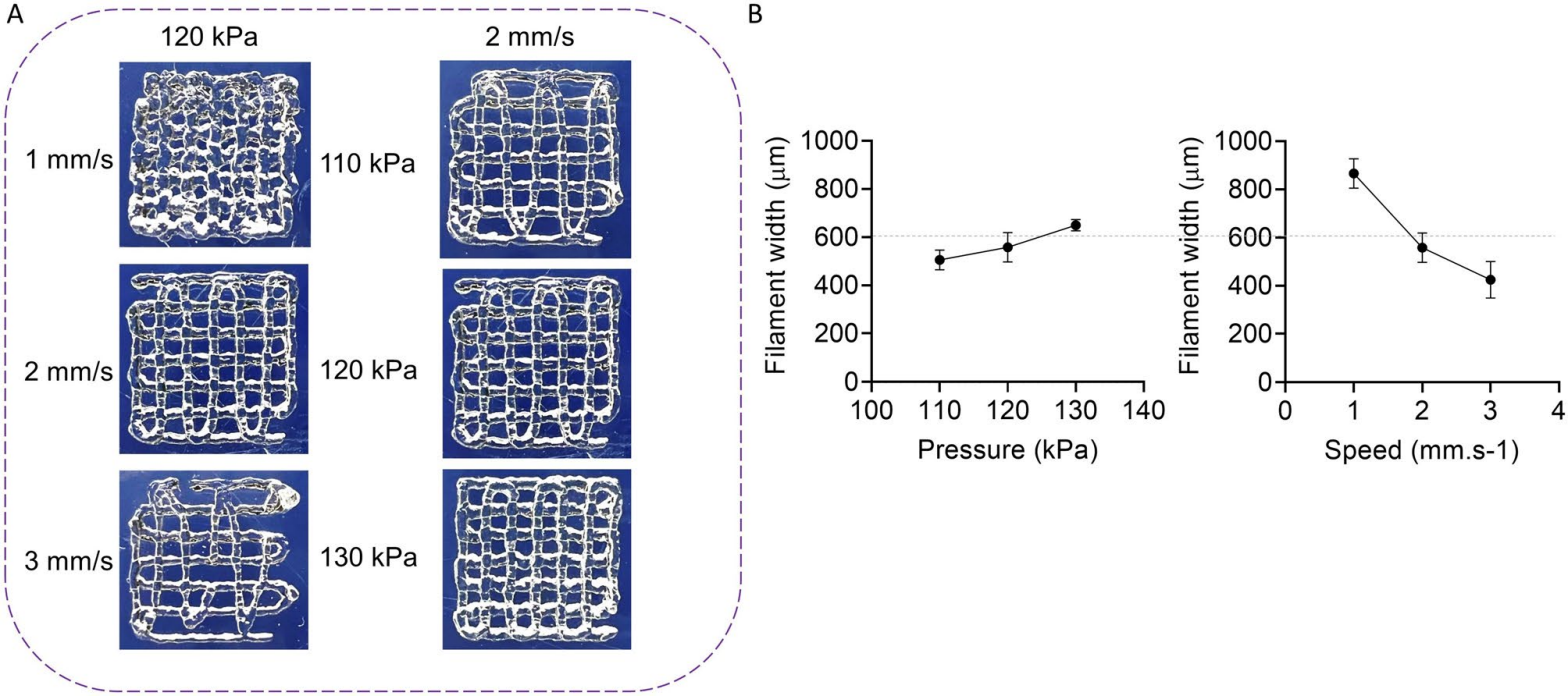
Effect of the printing parameters on the filament width. Pressure (110, 120 and 130 kPa) and speed (1, 2 and 3 mm/s) effects on the filament (**A**) printability and (**B**) width.

In a next step, we examined the effect of the printing pressure and speed on the filament width to identify the appropriate conditions for the fabrication of the final designs. We printed two layers of bioink at 3 pressures and 3 speeds (see Fig. 6A) and plotted the filament width versus pressure and speed (Fig. 6B). We note that Fig. 6 shows the data for printing with a 600 µm nozzle only, while we also repeated this step for 400 µm and 500 µm nozzles (data is not presented). To set the appropriate printing parameters, we used the nozzle inner diameter value as an ideal filament width and determined the suitable parameters from Fig. 6B through interpolation. It is noticeable that there is more sensitivity of filament width to speed rather than pressure and based on that, we chose a speed of 2 mm.s^–1^ and a pressure of 125 kPa for printing with a 600 µm nozzle. Similarly, we identified the suitable pressures to print filaments with diameters of 400 and 500 µm. The specified pressures were 180 and 150 kPa, respectively.

### Effects of the extrusion process on the mechanical properties of the printed construct

**Figure 7:**
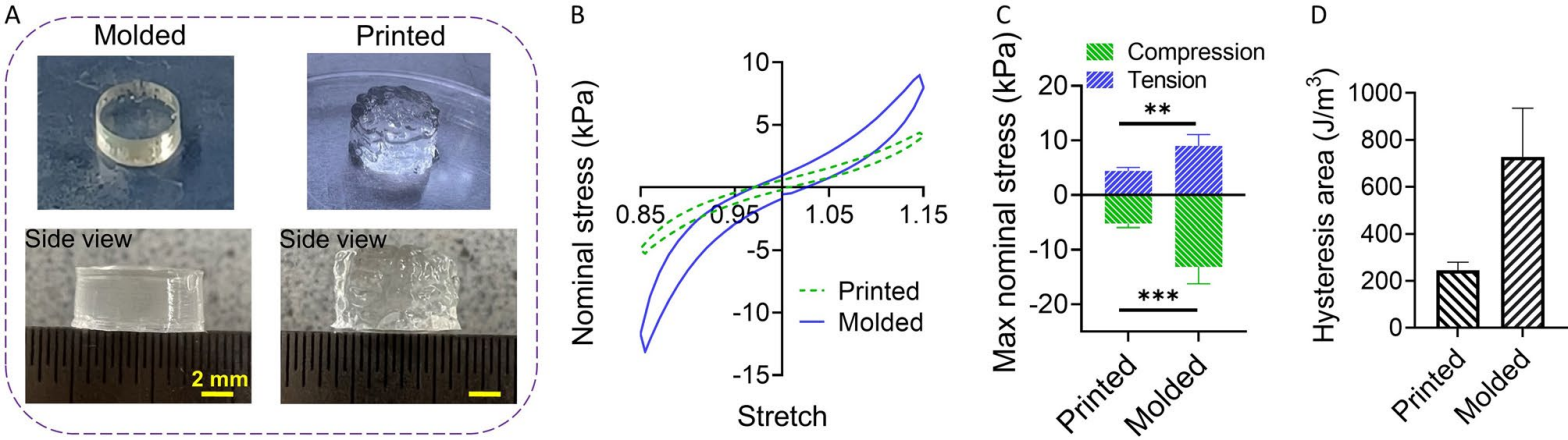
Comparison of mechanical properties of printed and molded samples: (**A**) fabricated samples; (**B**) stress-stretch behavior during the third cyclic compression-tension; (**C**) maximum nominal stresses in compression and tension; (**D**) hysteresis area, significance value **p < 0.01, ***p < 0.001 (n=5).

To investigate the effect of the extrusion process on the mechanical properties of constructs fabricated using the AG bioink, we prepared molded and printed samples with a similar protocol, as illustrated in Fig. 7A. This way, we aimed to assess the effect of the extrusion process, while keeping the effects of time and temperature to a minimum. We used a 600 µm nozzle for printing. Figure 7B shows the mechanical response of printed and molded samples during the third cycle of cyclic compression-tension loading. Printed samples show significantly softer mechanical responses in compression and tension, as well as a less pronounced hysteresis (area enclosed within the loading and unloading paths of the stress-stretch response) than molded samples (see Fig. 7C and D). As previously observed for AG hydrogels, the stress values in compression are higher than in tension^37^.

**Figure 8:**
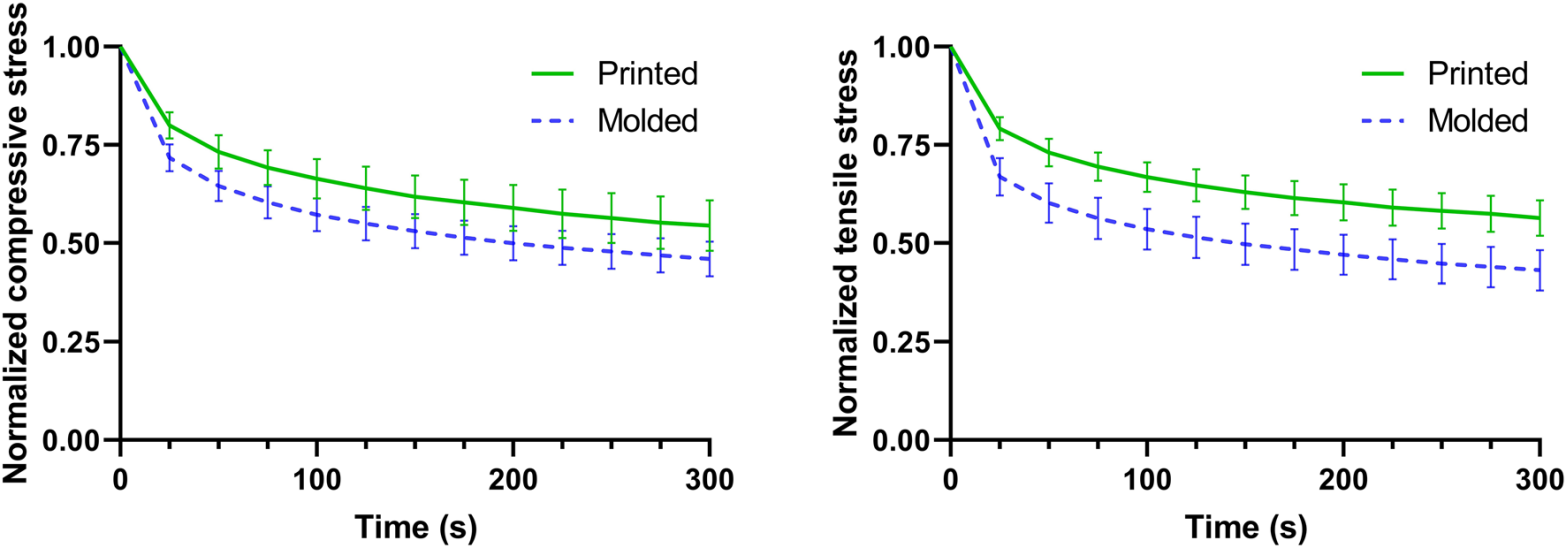
Normalized stress relaxation behavior of printed and molded samples in compression and tension (n=5).

In addition, we investigated the effect of the extrusion process on the relaxation behavior (see Fig. 8). Molded samples, where approximately 55% of the initial stress has relaxed after 300 seconds, are more viscous and respond faster in both compression and tension than printed samples, where only approximately 45% of the stress has relaxed after 300 seconds. This agrees well with the larger hysteresis areas during cyclic loading for molded samples in Fig. 7D.

### Effect of geometrical parameters on the mechanical properties of the printed construct

**Figure 9:**
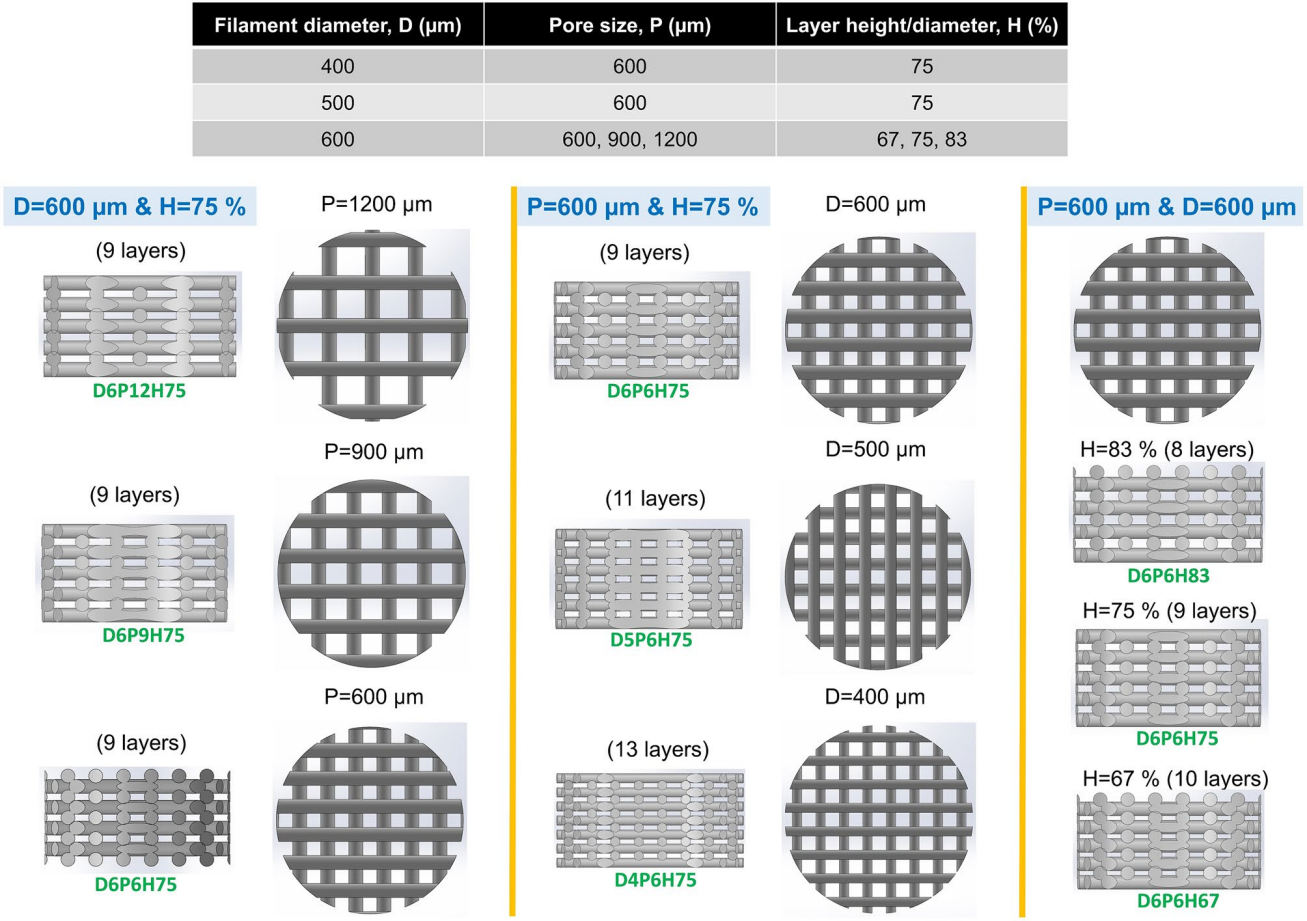
Design parameters and CAD models for printing different mesostructures using AG bioinks. The filament diameter, pore size and layer height were varied to obtain seven different structures.

After identifying suitable conditions and parameters for printing, we investigated the effects of geometrical parameters on the mechanical properties of printed constructs. The design parameters and CAD models for structures resulting in different macroporosities are summarized in Fig. 9. We designed seven patterns with three different filament diameters, pore sizes and layer heights. We printed the corresponding samples after cooling the AG bioink and then placing the nozzle at 25 °C for 60 mins according to the results of the previous steps. We used a maximum of 1.5 ml bioink. The printing parameters are summarized in Table 1.

**Table 1:** Printing parameters for fabricating different mesostructures using AG bioinks.

Sample type	Nozzle size (µm)	Pressure (kPa)	Speed (m.s^–1^)	Temperature (°C)	Layer height (µm)
D6P6H75	600	125	2	25	450
D6P9H75	600	125	2	25	450
D6P12H75	600	125	2	25	450
D5P6H75	500	150	2	25	375
D4P6H75	400	185	2	25	300
D6P6H67	600	125	2	25	400
D6P6H83	600	125	2	25	500

**Figure 10:**
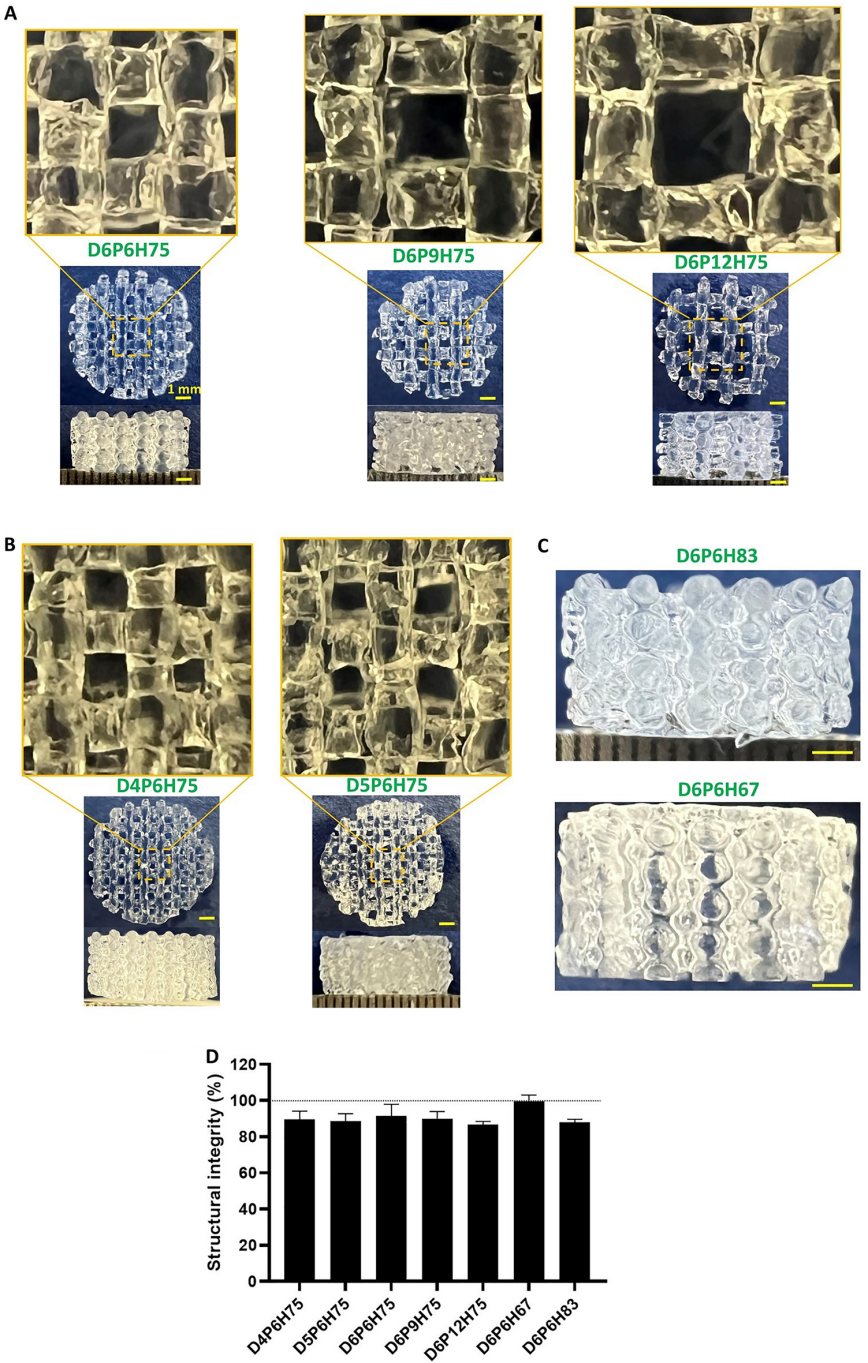
Optical images of seven print patterns and their structural integrity: (**A**) Different pore sizes of 600, 900 and 1200 µm; (**B**) Nozzle sizes of 400 and 500 µm; (**C**) Layer heights of 67 and 83% of the filament diameter (600 µm); (**D**) Structural integrity of different patterns (n=5).

Figure 10A–C shows images of the printed structures with different pore sizes, filament diameters, and layer heights. The corresponding quantified geometrical parameters are summarized in Table 2. It should be noted that we printed all samples with a printing speed of 2 mm/s but used different pressures for the nozzles of different diameters. As we noticed that for the identified pressure value for a 400 µm nozzle, the layers did not stick to each other, we decided to increase the pressure to 185 kPa. This allowed us to print the sample, but with an actual filament diameter that was higher than the designed one (483 µm compared to 400 µm in Table 2). Except for this sample, other samples were printed using the identified pressures and there was a high degree of similarities between the printed and designed filament diameters and porosities in most of the samples. We also calculated the structural integrity of the mesostructures, as shown in Fig. 10D. By increasing the layer height from 67 to 83% of the filament diameter (resulting in lower layer penetration), the structural integrity of the sample decreases from 99 to 88%. In addition, the structural integrity decreases from 91 to 87% when the pore size increases from 600 to 1200 µm.

**Table 2:** Filament diameters and porosities of different printed mesostructures in comparison with their designed values (n=5).

Sample type	CAD filament diameter (µm)	Printed filament diameter (µm)	CAD porosity (%)	Theoretical porosity (%)	Printed porosity (%)
D4P6H75	400	483.08±13.59	57.84	56.61	47.28±3.46
D5P6H75	500	527.96±40.17	52.61	51.04	50.90±4.50
D6P6H75	600	607.52±33.20	48.32	46.40	45.78±3.33
D6P9H75	600	610.48±23.74	57.59	56.23	57.82±3.44
D6P12H75	600	626.32±32.97	63.54	62.45	62.60±1.84
D6P6H67	600	626.52±54.44	43.88	40.78	40.15±6.53
D6P6H83	600	615.76±28.95	52.26	51.47	49.32±5.27

**Figure 11:**
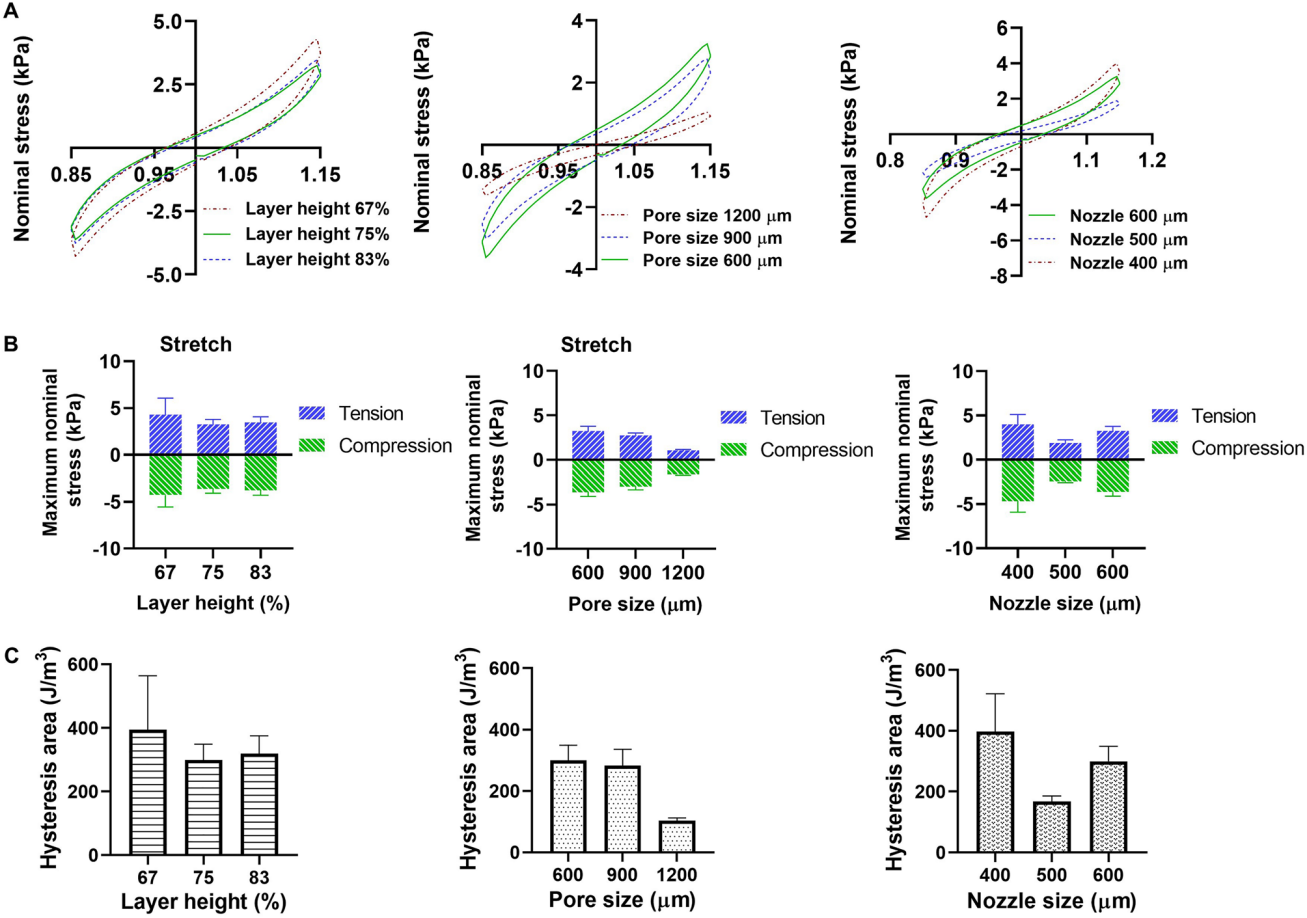
Cyclic compression-tension behavior of different printed structures: (**A**) Stress-stretch curves showing the effects of layer height, pore size and nozzle diameter (from left to right); (**B**) Maximum nominal stresses in tension and compression for different print patterns; (**C**) Hysteresis area (n=5).

In a last step, we characterized the effect of filament diameter, pore size, and layer height on the mechanical properties of the printed samples. Figure 11A shows the average conditioned response (third cycle) during cyclic compression-tension tests. The corresponding maximum nominal stresses in compression and tension as well as hysteresis areas are presented in Fig. 11B and C. The results show that the lowest layer height results in highest maximum stresses, which may be attributed to higher layer penetration. With increasing pore size, the maximum nominal stresses decrease as the porosity increases from 46 to 63% in Table 2. The trend regarding the nozzle diameter is less consistent, which could be associated with the higher filament size when printing structures with a 400 µm nozzle: As reported in Table 2, there is a high deviation of the printed porosity of the structures fabricated by 400 µm from its designed and theoretical porosities (47% compared to 58% and 57%, respectively). The trends regarding the hysteresis area for different geometrical parameters are similar to those of the maximum nominal stress.

**Figure 12:**
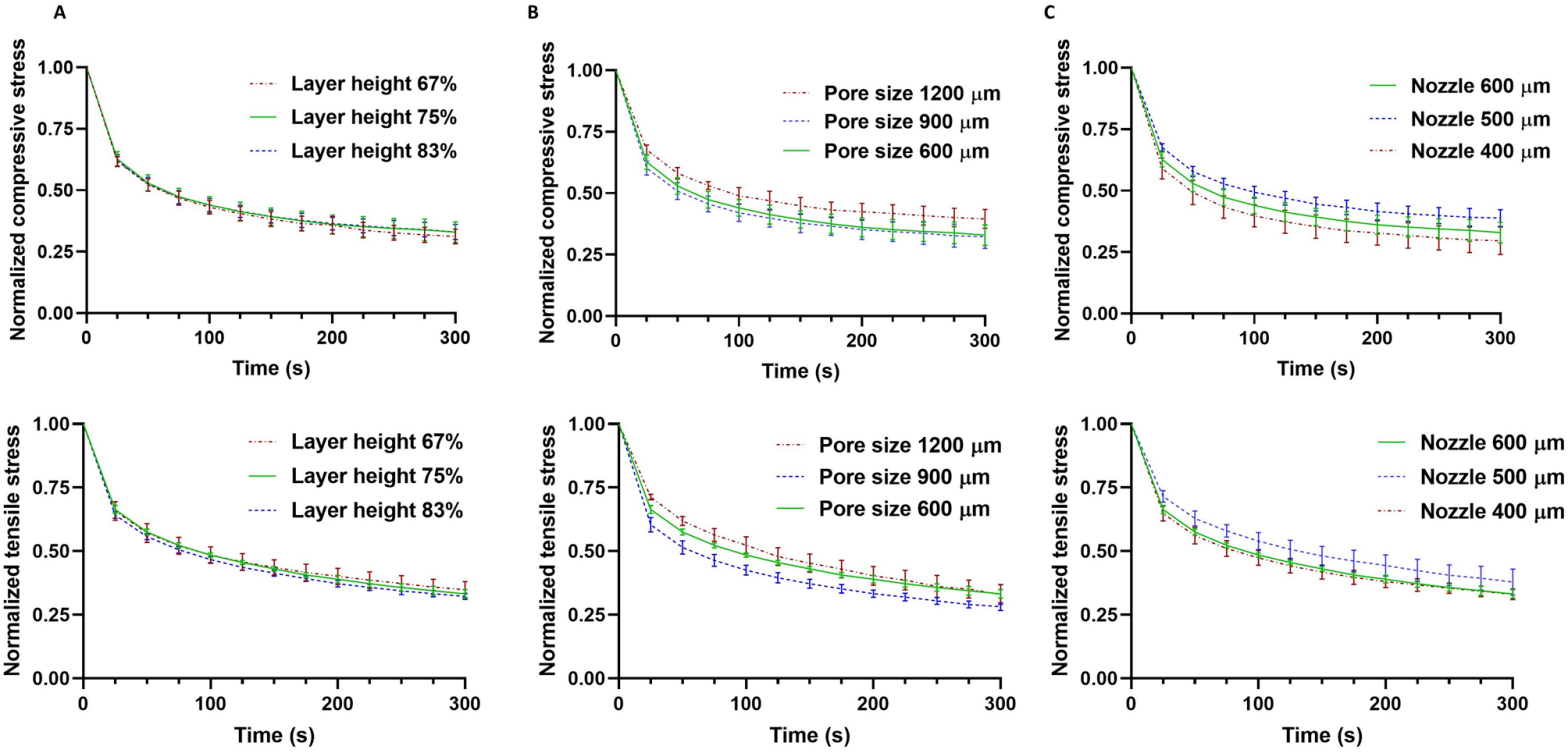
The effect of layer height (**A**), pore size (**B**), and nozzle size (**C**) on the relaxation behavior of different printed structures (n=5) in compression (top row) and tension (bottom row).

Figure 12 shows the relaxation behavior of the different printed structures in compression and tension. The layer height does not significantly affect the relaxation behavior in both compression and tension. Structures with 1200 µm pore size relax slower than structures with pore sizes of 600 and 900 µm, especially under tensile loading. This is in accordance with the smaller hysteresis area in Fig. 11C. Finally, structures printed with the 500 µm nozzle are less viscous than structures printed with 400 and 600 µm nozzles with less pronounced stress relaxation and smaller hysteresis area in Fig. 11C.

## Discussion

In this study, we have investigated the effect of the extrusion process and different mesostructures on the complex mechanical properties of 3D printed constructs. To successfully fabricate well-defined 3D structures, we have developed a protocol to print AG hydrogels with stable rheological properties, which has important implication for future 3D bioprinting applications. Especially due to the fact that bioprinting with embedded cells needs to be performed fast (less than 3 hrs) to prevent cell death in the syringe, the proposed cooling process will be essential. It accelerates the gelation of AG hydrogels and ensures more stable hydrogel properties when it flows out of the nozzle (Fig. 2)^29^. By carefully assessing the rheological property changes over time for different temperatures, we adapted the protocol to optimize the printing outcome. Our results show that 5 mins of cooling is sufficient and further increasing the cooling time does not affect the rheological properties (Fig. 3). In a previous study, the printability and structural integrity of GelMa bioinks was improved by using a similar pre-cooling step, as it led to thermo-crosslinking before printing^34^. However, this study focused on 2D porous and 3D non-porous structures without precise geometrical validations.

Our results have shown that the rheological properties are more stable at lower temperatures (Fig. 2), which can be attributed to the shorter gelation times at lower temperatures^29^. In turn, the printability increased with increasing temperature within the tested range of 23 °C, 24 °C, and 25 °C (Fig. 4B–D). Therefore, we chose 25 °C as the suitable temperature to print our constructs. This agrees well with a previous study, where a 1% w/v Alginate-5% w/v Gelatin bioink was printed at 25 °C and a good printability was achieved^29^. The high degree of stability against gravity of the AG bioink system used in the current study was also confirmed by the collapse test at different pressures (Fig. 5A).

Another property of the AG bioink that needed special attention was the highly unsteady mass extrusion rate within the initial 40 mins of extrusion at constant temperature and pressure (Fig. 5B). To address this issue and to ensure a steady extrusion rate and filament diameter, we started printing the final constructs only after this initial period. Without changing printing pressure and speed during fabrication, we were able to print structures that well resembled the designed models (Fig. 10). In addition, we identified the printing pressures and speeds that allowed us to print filament diameters similar to the nozzle diameters. As expected, the filament diameter increased with increasing pressure and decreasing speed, with more sensitivity to the printing speed. From these data, we identified the optimal printing parameters through interpolation. The procedure worked well for the nozzle sizes of 600 and 500 µm. However, for the 400 µm nozzle, we needed to slightly increase the pressure as we ran into the issue of insufficient layer bonding. This adjustment led to an increased filament diameter and geometrical deviation of the fabricated sample from the designed model (Table 2). Still, by changing the layer height or the printing speed, it was possible to improve the layer bonding, which could be further explored in the future.

In a next step, we studied the effect of the extrusion process on the complex mechanical properties of the final AG hydrogel constructs. Our results demonstrate that the fabrication process affects the cyclic compression-tension behavior, where printed samples show significantly lower maximum stresses than molded samples (Fig. 7C). This is in good agreement with a previous study, showing that the elastic modulus and compressive stresses (up to 7% strain) in molded samples were higher than in the printed samples^21^. This observation can be explained by the fact that layers are only partially bonded in printed samples in contrast to molded ones. For instance, a study investigating polylactic acid (PLA) constructs proved that the bonding between layers is not perfect in printed samples^4^. Our findings thus further highlight the importance of assessing the mechanical properties of printed not molded materials when designing biofabricated tissue-mimicking models. Our results also show that the energy dissipation in printed samples is lower than in molded samples. Accordingly, printed samples show smaller hysteresis areas and a less pronounced stress relaxation behavior than molded ones (Figs. 7D and 8), which can be attributed to the less densely packed material in printed than in molded samples – with less material contributing to the observed viscoelastic effects. Our observations have important implications for cell bioprinting as the viscous properties and stress relaxation behavior particularly affect the cell behavior^38,39^. To the best of our knowledge, this is the first time that 3D extruded AG hydrogels have been mechanically tested under large deformations.

In a final step, seven structures with different layer heights, nozzle, and pore sizes were printed to investigate the effects of these geometrical parameters on the mechanical properties of the printed constructs. Through the optimized procedure including the pre-cooling step, we achieved a high degree of similarity between the printed constructs and their design models, quantified through imaging and porosity measurements (Table 2). The only exception were the structures with a filament diameter of 400 µm. The structural integrity of our constructs ranged between 87 and 99% (Fig. 10D), which is in a good range based on previous recommendations^31^. Interestingly, the previous study also found that printing AG hydrogels with a tan (δ) between 0.25 and 0.45 resulted in high structural integrity. The tan (δ) of our cooled AG bioink at 25 °C was 0.4 (Fig. 2A). Finally, it was stated that a lower tan (δ) results in decreased printability, which again agrees with our findings, as lowering the temperature from 25°C to 23°C decreased the tan (δ) from 0.4 to 0.3, and led to worse printability (Fig. 4B–D). Our results indicate that the temperature affects the structural integrity, which could be more intensively studied in the future.

The designed and printed structures’ porosities were similar in fabricated samples using 500 and 600 µm nozzles, confirming a high degree of precision for these nozzle sizes with the chosen printing parameters. To the best of our knowledge, this is the first time that 3D structures with different geometrical properties have been printed using AG hydrogels as we managed to maintain a steady hydrogel flow during fabrication through the presented procedure. The filament diameters in printed samples were 608 to 627 and 527 µm with 1.3 to 4.5% and 5.6% deviation from their intended 600 and 500 µm values, respectively, demonstrating sufficient printing accuracy (Table 2). However, we had difficulties printing a layer height of 75% of the filament diameter (100 µm layer penetration) with a 400 µm nozzle at a printing speed of 2 mm/s, resulting in a 20.8% difference between the designed and printed filament diameter size. The increased filament diameter caused a highly increased layer penetration (183.2 µm or 83%) and higher stress values. These results show the high impact of the filament diameter value on the mechanical properties, which was also demonstrated in a related study using the Finite Element Method (FEM)^3^.

All structures showed viscoelastic properties and stress levels that were lower than for the bulk printed samples (Figs. 7C and 11B). Geometrical parameters affected the mechanical properties in both cyclic and relaxation compression-tension tests. The maximum compressive and tensile stresses were the highest in the samples with the lowest layer height (Fig. 11B). In addition, the average maximum stress decreased with increasing pore size in both tension and compression (Fig. 11B). Regarding the effect of the filament diameter, our data do not allow us to draw definitive conclusions. Although the maximum stresses were lowest in structures with a 500 µm filament diameter, the limitations when printing with the 400 µm nozzle might have affected the properties of the corresponding samples and led to an unrealistically stiff response (Fig. 11B). Previously, FEM simulations had suggested that the elastic modulus decreases with decreasing filament diameter for PLA constructs^40^.

Interestingly, the stress relaxation behavior appeared not to be affected by the layer height, i.e. variations of layer penetration from 100 to 200 µm (83 to 67% layer heights, respectively) did not lead to changes in the normalized stress (Fig. 12A). Regarding the effect of the pore size, we observed that the structures with 1200 µm pore size showed less stress relaxation than the other two structures in both compression and tension (Fig. 12B). Also, the hysteresis areas were smallest in these samples in Fig. 11C, which can explain the less pronounced stress relaxation. Therefore, we may conclude that changing the pore size affects the viscoelastic properties of printed constructs. Likewise, changing the filament diameter affects the relaxation behavior, where the structure with 500 µm filament diameter showed the smallest hysteresis area and least pronounced stress relaxation behavior (Figs. 11C and 12C). These geometrical effects and resulting viscoelastic properties could also influence the biological activity of cells in cell-laden bioprinted constructs.

## Conclusion

In this study, we have successfully printed multilayered structures with high control of geometrical parameters using a new procedure including a cooling process and optimized printing parameters based on rheological tests to enhance the printability of AG bioinks. We have demonstrated that the extrusion process significantly changes the mechanical properties of the printed hydrogel constructs. Different 3D printed structures with varying layer height, pore size, and filament diameters (resulting in different mesostructures and macroporosities) yielded distinct mechanical properties – with maximum stresses ranging from 1.05 to 4.23 kPa in tension and 1.62 to 4.69 kPa in compression as well as altered stress relaxation behavior. Our results highlight the importance of geometrical properties in modulating the mechanical behavior of bioprinted constructs with important implications for the future design of mimicking materials for different tissues with varying mechanical properties, especially for soft tissue engineering applications. The presented methodology for preparing and printing AG hydrogels is also compatible with cell bioprinting approaches and can be used in the future to print precise cell-laden structures with different geometries and mechanical properties.

## Data Availability

The datasets generated and/or analyzed during the current study are available from the corresponding author on reasonable request.
